# Growth of a deep-water, predatory fish is influenced by the productivity of a boundary current system

**DOI:** 10.1038/srep09044

**Published:** 2015-03-12

**Authors:** Hoang Minh Nguyen, Adam N. Rountrey, Jessica J. Meeuwig, Peter G. Coulson, Ming Feng, Stephen J. Newman, Anya M. Waite, Corey B. Wakefield, Mark G. Meekan

**Affiliations:** 1 School of Animal Biology, University of Western Australia, Crawley WA, Australia; 2 Centre for Marine Futures, Oceans Institute, University of Western Australia, Crawley WA, Australia; 3 Museum of Paleontology, University of Michigan, Ann Arbor MI, USA; 4 Centre for Fish and Fisheries Research, School of Veterinary and Life Sciences, Murdoch University, Murdoch WA, Australia; 5 CSIRO Oceans & Atmosphere Flagship, Underwood Avenue, Floreat, WA 6014, Australia; 6 Western Australian Fisheries and Marine Research Laboratories, Department of Fisheries, Government of Western AustraliaPO Box 20, North Beach, WA 6920, Australia; 7 School of Civil, Environmental and Mining Engineering, University of Western Australia, Crawley WA, Australia; 8 Australian Institute of Marine Science, Crawley WA, Australia

## Abstract

The effects of climate change on predatory fishes in deep shelf areas are difficult to predict because complex processes may govern food availability and temperature at depth. We characterised the net impact of recent environmental changes on hapuku (*Polyprion oxygeneios*), an apex predator found in continental slope habitats (>200 m depth) by using dendrochronology techniques to develop a multi-decadal record of growth from otoliths. Fish were sampled off temperate south-western Australia, a region strongly influenced by the Leeuwin Current, a poleward-flowing, eastern boundary current. The common variance among individual growth records was relatively low (3.4%), but the otolith chronology was positively correlated (r = 0.61, p < 0.02) with sea level at Fremantle, a proxy for the strength of the Leeuwin Current. The Leeuwin Current influences the primary productivity of shelf ecosystems, with a strong current favouring growth in hapuku. Leeuwin Current strength is predicted to decline under climate change models and this study provides evidence that associated productivity changes may flow through to higher trophic levels even in deep water habitats.

Demersal fishes that occupy relatively deep waters (around 200 m) at the edge of the continental shelf are likely to be particularly vulnerable to the alterations in marine ecosystems predicted to occur under scenarios of climate change in the near future (50–100 years). In part, this vulnerability is due to strong associations with the seabed that may constrain their ability to move around barriers created by inappropriate habitats (see Part C in reference 1). They are also thought to be at risk because such species tend to be targets of major commercial fisheries, so that in many cases, their populations are already heavily exploited^1^,^2^,^3^,^4^. Thus, climate change may act on species that already have populations with limited resilience due to over-harvest.

Our ability to predict the responses of deep water fishes to climate change is complicated by their trophic role as secondary carnivores or apex predators. For such species, productivity input into the ecosystem can be modified by a complex web of interactions and energy flows occurring in lower parts of the food chain that can take place remotely, in pelagic or shallow water ecosystems^5^,^6^. Furthermore, species in deep water are removed from the direct effects of changes in some physical variables such as sea surface temperature. The difficulty in determining the likely effects of climate change on deep water fishes has been acknowledged as a major challenge for research (as reviewed in Ref. 7).

Although there is a growing number of studies e.g.^8^,^9^,^10^,^11^,^12^ that examine how increasing sea surface temperatures may affect the distribution and abundance of marine fishes, climate change may also lead to modification of ocean circulation and changes in the primary productivity of the oceans^13^,^14^,^15^. Simultaneous changes to multiple aspects of marine systems mean that it is difficult to predict net impacts and to envisage how these might influence individual-level variables, such as growth rate, which will integrate this complex interplay of drivers. In part, this is due to a lack of long-term (decadal) observational data sets sufficient to investigate the relationships between vital rates of fishes in natural environments and variation in climate.

Recent studies show that growth records from otoliths may be particularly useful in filling this knowledge gap^16^. This research applies techniques developed in dendrochronology (the study of tree-rings) to data extracted from otoliths so that growth records from many fish can be aligned and combined into a single time-series^17^. This allows enhancement of the population “signal” (i.e. the common growth response to regional environmental drivers, such as temperature, productivity and current flow) above individual variation.

Here, we use a dendrochronology approach to construct and analyse long-term (decadal) growth records of the hapuku (*Polyprion oxygeneios*), a large (up to 175 cm) predatory teleost typically found at the shelf edge in depths of 200–850 m^18^,^19^. Our focus is on individuals collected from a region of relatively rapid warming off the south-west of Western Australia^20^,^21^. Not only is the region warming, but models suggest that there will be substantial changes to regional oceanography in this century, including weakening of the Leeuwin Current^22^,^23^, which exerts a major influence on the productivity of the coast of WA^22^. We identify key environmental drivers of growth rate for this apex, deep-water predator and consider the implications of environmental conditions predicted to occur in the near future (within 50 years) on the growth of this species.

## Results

Our sample included fish from 27 to 49 years of age. A spline rigidity with a 50% frequency cut-off of nine years (see Supplementary Notes S1) was selected for standardizing measurements from transects before averaging transects for each individual. The mean sensitivity (a measure of high frequency variation, see Ref. 24) of individual mean series (IMS) was 0.14, indicating a relatively low degree of interannual variation in increment widths.

**Figure 1:**
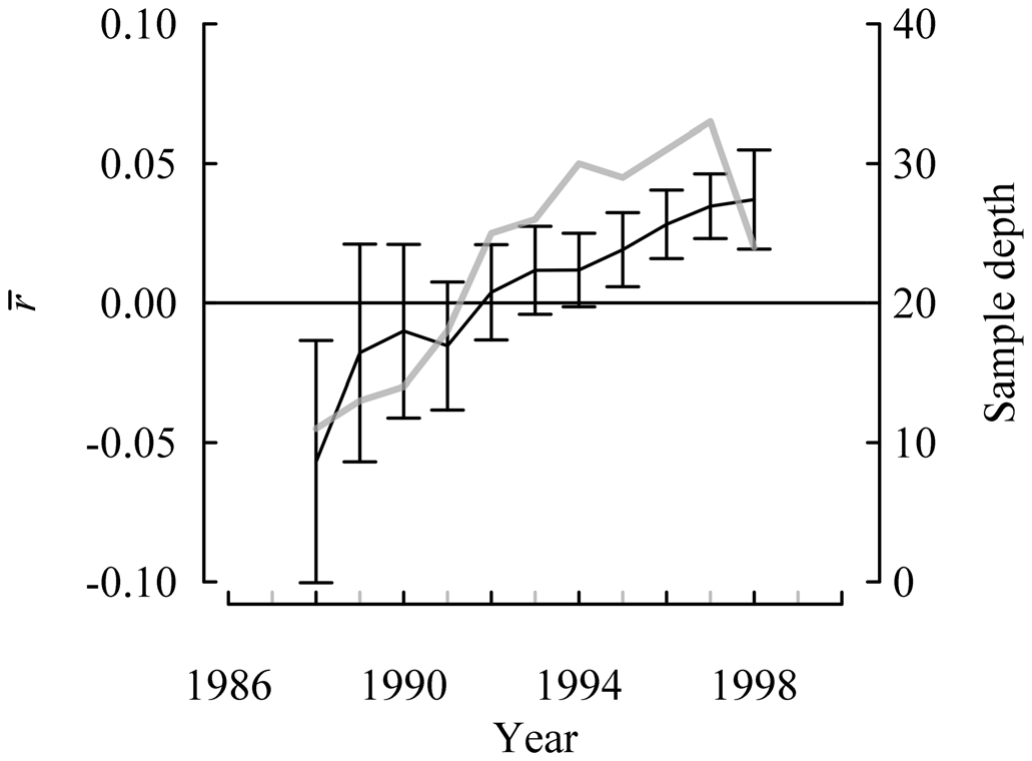
Pairwise correlations among all series excluding self-correlations (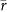) ± s.e.(black line) and number of individual 15-year series contributing to the calculation (sample depth, grey line). Values are plotted at the centres of the 15-year intervals. The 1997 (i.e., 1990–2004) segment was used to assess the influence of environmental factors.

**Figure 2:**
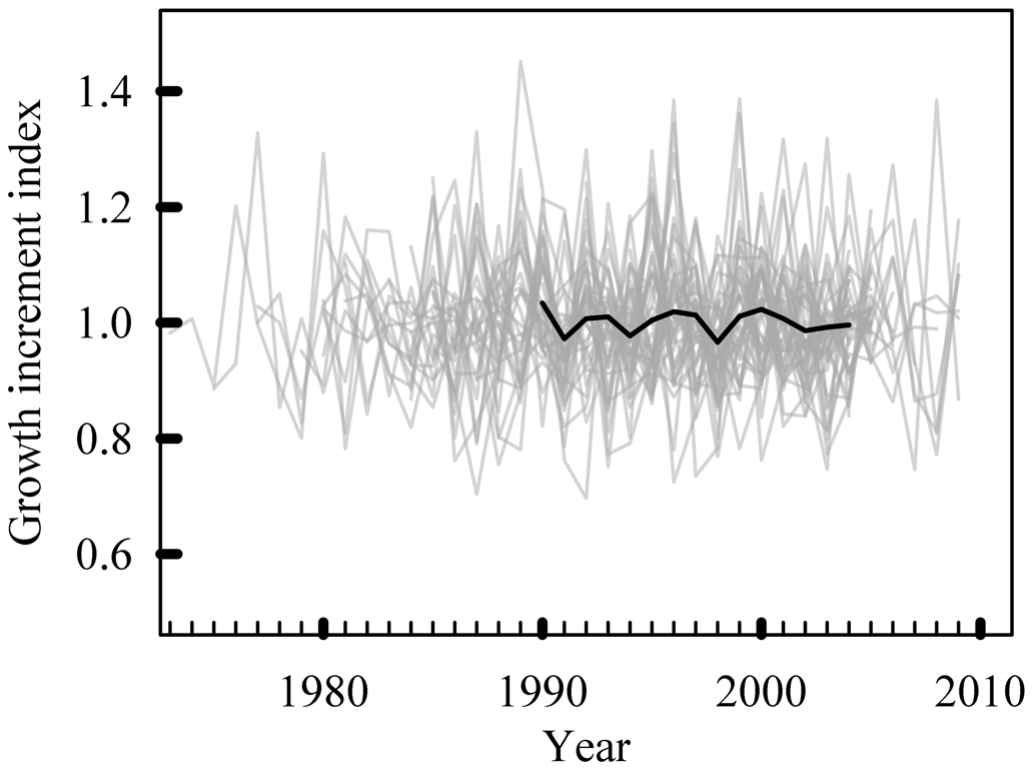
Individual mean series (IMS, grey lines) of otolith growth for the 44 hapuku after detrending using a spline with a 50% frequency cut-off of nine years, and the mean index chronology (MIC, black line) covering the selected 15-year period.

In order to identify a segment of the mean index chronology (MIC) with adequate signal representation, we used the criterion that the value obtained from subtracting two times the standard error from 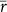 (the mean of the pairwise correlations among all series excluding correlations of series with themselves) must be greater than 0 for a 15-year segment. The 15-year periods of 1989–2003, 1990–2004 and 1991–2005 had 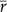 – 2 s.e. greater than 0 (Fig. 1), and we chose to focus on the period of 1990–2004 (Fig. 2) because a larger number of pairwise correlations were included in the 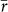 calculation relative to other segments. The expressed population signal (EPS, see Supplementary Notes S1) for the 1990–2004 period was 0.60 with a 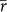 of 0.035 (s.e. = 0.012). The MIC for this period was used in all subsequent analyses.

Correlation tests with environmental variables revealed significant correlations (p < 0.05) with the Multivariate El Niño/Southern Oscillation Index (MEI) (r = −0.62), Fremantle sea level (r = 0.61), and sea surface temperatures in austral autumn, winter, and spring of the previous year (Table 1). There were no significant correlations with variables in the year of growth (Table 1).

## Discussion

### The Leeuwin Current as a driver of growth of a deep-water predator

Growth of hapuku (as measured by the mean index chronology from 1990–2004) was positively correlated with the previous year's mean annual sea level at Fremantle, which is a proxy for the strength of the Leeuwin Current through El Niño-Southern Oscillation cycles^25^,^26^. Given that this current exerts a major influence over sea surface temperature and productivity of shelf waters off south-western Australia^22^, this correlation likely reflects a relationship between growth of this species and a number of oceanographic/ecological processes occurring within the Current system. In austral autumn and winter, the Leeuwin Current intensifies and produces mesoscale (2–100 s of km) eddies^27^ that spin up just beyond the shelf break^28^,^29^,^30^. Warm core eddies increase the instability of the Current and destratify the water column, flooding the shelf-break with relatively nutrient-rich waters sourced from the shelf itself^29^ during austral autumn and winter (March–August). Cold-core eddies move deep nutrient-rich water to the surface via upwelling^27^. Both warm and cold core eddies therefore contribute an offshore component to the regional increase in primary production commencing in March and peaking in May^27^,^29^,^31^.

The delay between the response of hapuku growth in one year and the period of peak productivity induced by the Leeuwin Current in the preceding May^26^,^29^ is probably caused by the transfer time required for increases in productivity to reach upper trophic levels of the food chain upon which hapuku prey. Analyses of gut contents of hapuku indicate that squid (most likely arrow squid, *Nototodarus gouldi*, S. Leporati pers. comm.) are a major (>50%) component of the diet (C. Wakefield pers. comm.;^32^). Squid typically show strong diel cycles of vertical migration, ascending at night to the thermocline and surface waters to feed on invertebrates and small fishes and descending to deep waters during the day^33^. This pattern of migration allows squid to form part of a relatively simple pelagic food chain, so that energy flows to deep apex predators are likely to be far more direct and rapid than if they were to pass through more complex benthic habitats. Squid also have relatively short life spans and their growth rates can respond very rapidly to changes in ocean conditions, with increased growth occurring in association with increased productivity and temperature^34^. Thus, squid may allow for fast transfer of productivity from the surface to large, deepwater predators. The lag between increased hapuku growth and the proximate impacts of a strengthened Leeuwin Current is likely to reflect the time required for prey to attain the sizes where they form a substantial part of the hapuku diet.

Hapuku growth was negatively correlated with MEI of the previous year and positively correlated with SST in the previous year (Table 1). The negative correlation with MEI, which indicates higher growth rates following La Niña years, is not surprising given that the Leeuwin Current is stronger during La Niña years^26^. Positive correlations of hapuku growth with SST in the previous year reflect the strong link between the Leeuwin Current and SST. It is unclear how direct effects (e.g. physiological) of environmental temperature could produce substantially lagged growth responses, and other studies have not shown such lags^35^,^36^,^37^,^38^. This supports the hypothesis that the growth of hapuku was driven by changes in productivity of the system (for which a lag would be expected), rather than by the direct effects of changes in water temperature.

### Hapuku growth and climate change

Ocean temperatures in Western Australia are predicted to rise by 0.5 to 1.0°C at depths down to 500 m, according to the CSIRO's Mk 3.5 climate change model and the atmospheric CO_2_ scenario of 536 ppm by 2050 (See Parts A and B in Ref. 1). While this moderate increase in temperature may have some direct impact on the growth of deep-dwelling fish like hapuku, it is possible that the larger impacts of climate change will eventuate through decreases in productivity associated with changes in regional oceanography. Subsurface cooling in the Western Equatorial Pacific, along with weakened Pacific trade winds and increased sea surface salinity, suggest that the strength of the Leeuwin Current will decrease over the next 100 years^23^,^30^. Downscale modelling from the CSIRO mark 3.5 model by Sun, et al.^39^ shows that these declines in current strength could be as great as 15% by the 2060s, with the largest decreases occurring in austral winter. Given the link between enhancement of primary productivity induced by the Leeuwin Current and the growth of hapuku, our study suggests that growth rates may decrease in the future, potentially impacting the fishery.

In summary, our study provides evidence of a link between the growth of deep water, predatory fishes and regional oceanographic processes. It contrasts with other work that has largely focused on the direct effects of changes in SST on the distribution and abundance of fishes e.g.^10^,^35^,^37^. The strength of the relationship between growth and the Leeuwin Current that we described may be a consequence of the trophic role of hapuku as a higher-order predator, with a diet that focuses on fast-growing forage species with strong links to relatively simple food chains in the pelagic environment that in turn rely on regional primary productivity. This suggests that other deep-water species that rely on squid or other vertically migrating prey in the region may show similar responses to variations in Leeuwin Current strength.

Our study shows that otolith biochronologies provide a relatively simple means to determine the drivers of growth of marine fishes in deep water on the shelf slope that are remote from some of the other physical effects of climate change such as warming sea-surface temperatures. These biochronologies can be used to examine relative influence on growth of both physical and biological factors, even when the study species is a high-order predator at the upper end of the food chain.

## Methods

### Study species

Hapuku have a circumglobal distribution between 28°S and 43°S, and individuals are typically found between depths of 50–450 m^18^,^40^, with most animals typically occurring at depths greater than 200 m. Adult hapuku range in size from around 70 cm to over 175 cm in total length^19^. The species is classified as an ‘opportunistic generalized carnivore'^41^ and has an unusual life history in that juveniles have a protracted (3–4 years) pelagic stage during which they associate with flotsam^42^. This stage provides a potential mechanism for long-range mixing among populations separated by thousands of kilometres.

Hapuku are long-lived, with a maximum observed age of 63 years for fish in New Zealand waters and 52 years for fish in waters off south-western Australia^19^,^42^, although fish over 20 years of age are relatively uncommon^42^. Their long lifespan enables compilation of a growth chronology covering decades using individuals captured over just several years. The age of maturation (for 50% of individuals) of both sexes is around seven years and spawning occurs from May to September in south-western Australia^19^. Growth rates of juveniles and adults are highest between January and May, although growth occurs throughout the year at lower rates^19^.

### Otolith analysis

**Figure 3:**
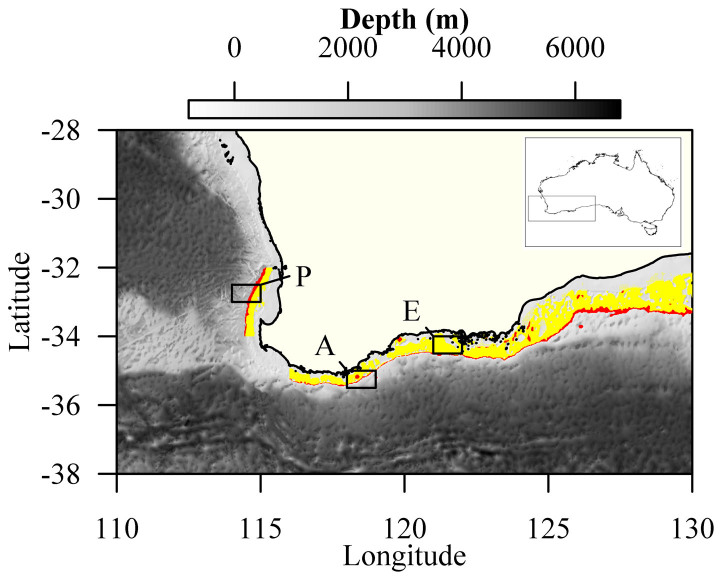
Southwestern Australia showing areas in which study fish were caught. Red highlighting indicates areas of the shelf corresponding to the depth of capture (200–450 m); yellow highlighting indicates other areas of the shelf with appropriate depth range for the species (50–200 m). Some localized deep areas on the southern shelf may represent errors in bathymetry data. Black rectangles mark grid cells for which sea surface temperature was extracted- P = “Perth”, A = “Albany”, E = “Esperance”. Inset shows location of detail map. Figure 3 was generated using R vers. 2.15.2 (R Foundation for Statistical Computing, Vienna, Austria), the PBSmapping package vers. 2.67.60^57^ and the marmap package^58^. The bathymetric data were from the global, self-consistent, hierarchical, high-resolution geography database (GSHHG) vers. 2.2.0^59^ and the coastal data NOAA 1 Arc-Minute Global Relief Model^60^.

Hapuku otoliths were obtained from the collections of the Department of Fisheries of Western Australia. Otolith sections (see Ref. 19) were produced from fish caught between 2005 and 2011 by commercial fishers employing multi-hooked vertical drop lines in 200 to 450 m waters off the south (115.5°E to 129.0°E) and south-west (32.0°S to 34.0°S) coasts, respectively (Fig. 3). Otoliths from the 50 oldest individuals were selected for analysis to maximize temporal coverage. Sections were scanned using transmitted light on an Aperio Scanscope™ (Aperio Technologies Inc., Vista, California, USA). Six sections were excluded due to issues associated with increment clarity.

**Figure 4:**
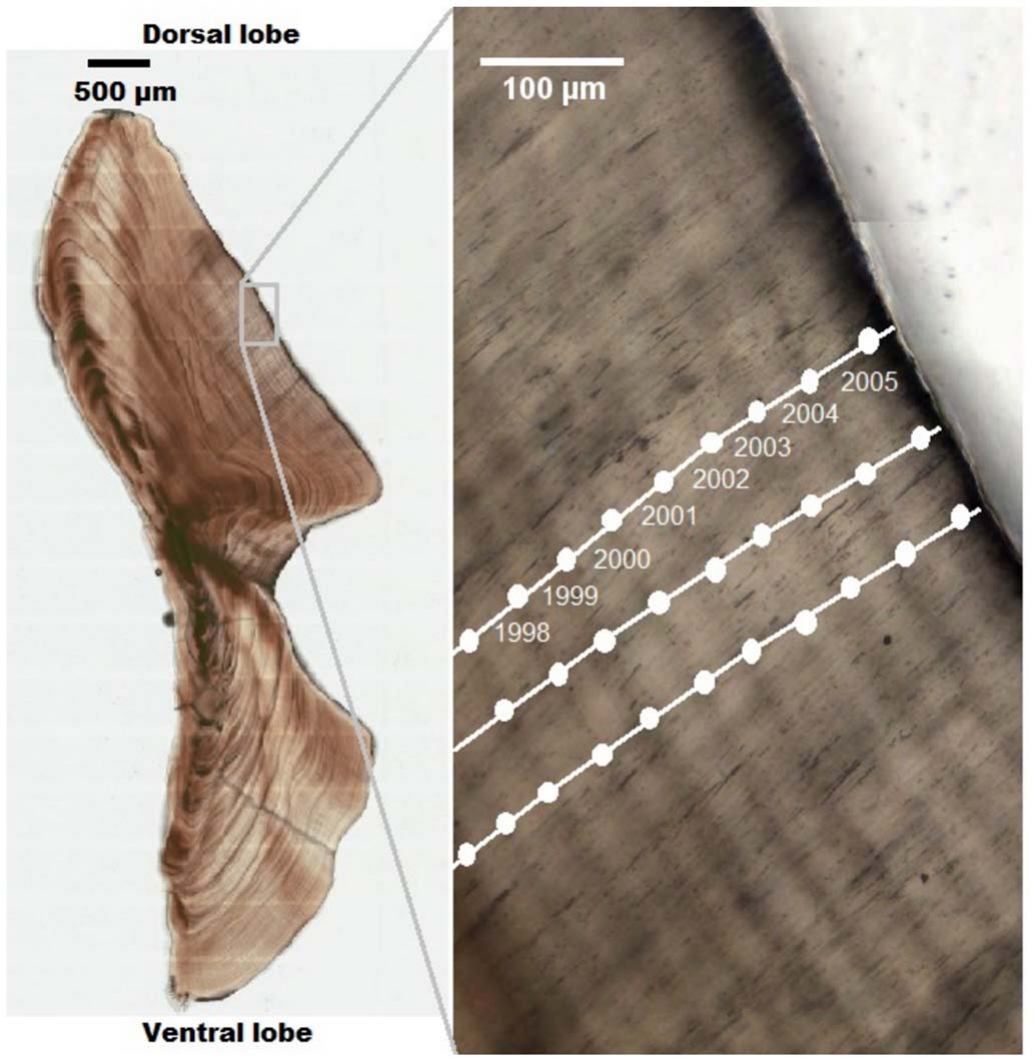
Transmitted light image of a sectioned otolith from a 32-year-old hapuku. The magnified section shows the dorsal mid-lobe region where opaque zones were marked (white circles) and increments were measured along three transects (white lines). Numbers identify the year in which the increments were formed.

Increment widths were measured using a polyline-transect-based measurement plugin^43^ for ImageJ vers. 1.45 s (U.S. National Institute of Health, Bethesda, Maryland, USA). Beginning at the periphery of the otolith and moving toward the primordium, a polyline transect was placed such that the line segments remained locally parallel to the direction of growth, i.e. perpendicular to the increment pattern (Fig. 4). Marks were placed at the middle of each opaque zone. Partial years at the outermost edge of the otolith section were excluded from the measurement series.

Increments were measured along three to five transects in each otolith to increase reliability. While it was possible to use the whole otolith section to count all opaque zones from the periphery to the primordium to establish ages for each individual, it was not possible to precisely measure the widths of all growth increments due to the unclear boundaries of increments corresponding to the first 10–12 years of life, a time that included changes in morphology associated with the transition from pelagic to deep water habitats^44^. For this reason measurements were restricted to increments formed after the age of 12 so that all measured increments represented growth in sexually mature individuals^19^.

### Biochronology development

A mean index chronology (MIC) was developed from the chronologies of the 44 fish by firstly crossdating, which aided the alignment of increment data to calendar years using the program COFECHA^45^. Chronologies were then detrended using R vers. 2.15.2 (R Foundation for Statistical Computing, Vienna, Austria) and the dplR package (dendrochronology program library in R)^46^,^47^ with some additional scripts. This involved removing ontogenetic trends and averaging the resulting series to increase the signal-to-noise ratio^48^. Initially, the increment measurement series from each transect was detrended (standardized) by dividing the series by a cubic smoothing spline with a 50% frequency cut off^49^,^50^ of 22 years^17^,^37^. For each fish, an individual mean series (IMS) was calculated by averaging the standardized indices from the three or five transects. The Pearson's correlation coefficient between each IMS and the mean of all other IMSs was calculated. The mean of those correlation coefficients is the interseries correlation, a measure of the strength of the common signal. Additional details on this procedure and the process used to select a final detrending scheme can be found in the Supporting Information Note S1. After crossdating and detrending, the program ARSTAN (Autoregressive Standardization)^48^ was used to obtain a MIC that accounted for autocorrelation.

Several environmental variables and climate indices were compiled in order to determine their potential influence on hapuku growth (Table 2). Pearson's correlation coefficients were used to investigate the relationship between the MIC and annual or seasonal means of these variables and indices. As the growth of an annual increment in an otolith represents the growth that occurred from approximately November through October (the “growth year”)^19^, increment measurements were assigned to the calendar year in which most growth represented in that increment occurred. We refer to this as the “current calendar year”. For the correlation tests, we included environmental values from the current calendar year and the previous calendar year to allow for the detection of the effects of the prior year's environmental conditions on growth. For gridded environmental datasets, we selected grid cells close to three locations along the lower west and south coasts of Western Australia: offshore Perth (−32.5°, 114.5°), Albany (−35.5°, 118.5°), and Esperance (−34.5°, 121.5°) (Fig. 3).

## Author Contributions

J.J.M., M.G.M., S.J.N. and H.M.N. conceived the study. H.M.N., A.N.R., P.G.C., J.J.M., M.G.M., S.J.N. and C.B.W. contributed to study design, material collection, laboratory work, data analysis and manuscript preparation, editing and improvement. M.F. and A.M.W. contributed to the data analysis, discussion, manuscript editing and improvement.

## Supplementary Material

Supplementary Information Supplementary Information

## Figures and Tables

**Table 1:** Correlations between the 1990–2004 mean index chronology and environmental variables. “Previous year” indicates the correlation between the 1990–2004 mean index chronology and environmental data from 1989–2003. Significant correlations are bolded and indicated by asterisks

Variable	Season	Current year *r*	*p*	Previous year *r*	*p*
Detrended annual Fremantle sea level	-	0.23	0.415	*0.61	**0.016**
Annual Multivariate ENSO Index	-	−0.19	0.500	*−**0.62**	**0.014**
Annual Dipole Mode Index	-	−0.35	0.195	−0.21	0.445
"Perth" sea surface temperature	DJF	0.42	0.120	0.018	0.948
	MAM	0.02	0.941	*0.62	**0.015**
	JJA	−0.33	0.236	0.47	0.080
	SON	0.14	0.621	0.51	0.053
"Albany" sea surface temperature	DJF	0.49	0.062	0.15	0.591
	MAM	0.32	0.246	0.46	0.086
	JJA	0.25	0.371	*0.61	**0.015**
	SON	−0.18	0.527	*0.6	**0.017**
"Esperance" sea surface temperature	DJF	0.21	0.447	−0.19	0.502
	MAM	0.38	0.158	0.18	0.510
	JJA	−0.04	0.879	*0.56	**0.029**
	SON	0.07	0.799	0.4	0.137

**Table 2:** Summary of environmental variables used in correlation tests. *Abbrev.*: lat: latitude; lon: longitude; Per: Perth; Alb: Albany, and Esp: Esperance. *Sources*: Met Office Hadley Centre^51^; BOM: Bureau of Meteorology, Australia^52^; JAMSTEC: Japanese Agency for Marine-Earth Science and Technology^53^; NOAA/PSD: National Oceanic and Atmospheric Administration/Physical Science Division: Earth System Research Laboratory^54^

Environmental Variable	Units	Temporal resolution	Grid Type	Grid Cell Locations			Source
				Loc	Lat	Lon	
Sea surface temperature (HadISST)	°C	seasonal means	1°	Per	−32.5	114.5	Met Office Hadley Centre
				Alb	−35.5	118.5	
				Esp	−34.5	121.5	
Fremantle sea level (linear detrended)	m	annual mean	-	-	-	-	BOM
							
							
Indian Ocean Dipole (IOD)^55^	-	annual mean	-	-	-	-	JAMSTEC
							
							
Multivariate ENSO Index (MEI)^56^	-	annual mean	-	-	-	-	NOAA/PSD
							
							
