# Supplementary material for: Growth of a deep-water, predatory fish is influenced by the productivity of a boundary current system
Source: Sci Rep. 2015 Mar 12;5:9044. doi: 10.1038/srep09044 (PMC4356959; doi:10.1038/srep09044)
Supplement: Supplementary Information [file srep09044-s1.pdf]

Growth of a deep-water, predatory fish is influenced by the productivity of a boundary current system

Hoang Minh Nguyen[1], Adam N. Rountrey[2,3], Jessica J. Meeuwig[1,2], Peter G. Coulson[4], Ming Feng[5], Stephen J. Newman[6], Anya M. Waite[7], Corey B. Wakefield[6] & Mark G. Meekan[8]

[1] School of Animal Biology, University of Western Australia, Crawley WA, Australia, [2] Centre for Marine Futures, Oceans Institute, University of Western Australia, Crawley WA, Australia, [3] Museum of Paleontology, University of Michigan, Ann Arbor MI, USA, [4] Centre for Fish and Fisheries Research, School of Veterinary and Life Sciences, Murdoch University, Murdoch WA, Australia, [5] CSIRO Oceans & Atmosphere Flagship, Underwood Avenue, Floreat, WA 6014, Australia, [6] Western Australian Fisheries and Marine Research Laboratories, Department of Fisheries, Government of Western Australia, PO Box 20, North Beach, WA 6920, Australia, [7] School of Civil, Environmental and Mining Engineering, University of Western Australia, Crawley WA, Australia; ***Current Address***: Alfred Wegener Institute Helmholtz Centre for Polar and Marine Research, Building E-2155, Am Handelshafen 12, D-27570 Bremerhaven, Germany, [8] Australian Institute of Marine Science, Crawley WA, Australia

Supporting information

Note S1 – Crossdating and detrending

Potential errors in growth increment marking were identified by statistical crossdating using the program COFECHA (i.e. crossdate in Spanish; [1]). Among other functions, this program performs a windowed cross-correlation analysis. In this analysis, the cross correlations (at lags -10 to 10 years) between segments of each individual mean series (IMS) and segments of the mean index chronology (MIC) (excluding the IMS of interest) were calculated using a 15-year moving window. A low positive or any negative correlation of a segment at zero lag indicated possible errors in the marking of increments, and high correlations at lags other than zero indicated alternative alignments. When the correlation at zero lag was low or negative, the otolith image and marked transects were checked visually, and if appropriate, errors were corrected.

Detrending of otolith increment series is often accomplished using a spline with a 50% frequency cut-off at 22 years or a negative exponential function[2-6]. Because we only measured growth increments formed after maturity, the negative exponential function, which accommodates rapid declines in increment thickness early in life, was not used. To select appropriate spline rigidity for detrending, we systematically explored a range of rigidities to determine the effects on chronology quality. In this case, optimal detrending should maximize the mean of the pairwise correlations among all series (excluding correlations of series with themselves), referred to as $\bar{r}$, while avoiding the introduction of artifactual correlations (e.g., those due to alignment of ontogenetic trends in multiple individuals). A commonly-used measure of chronology quality is the expressed population signal (EPS) which depends on $\bar{r}$ and the number of individual series analyzed (t) [7,8]:

$$EPS(t) = \frac{t \times \bar{r}}{t \times \bar{r} + (1 - \bar{r})}$$

The EPS value can range from 0 to 1 with a higher EPS indicating that the MIC better represents the hypothetical chronology that would result from including the entire population in the sample [8]. A higher EPS also indicates a better reconstruction of the statistical link between climate and the mean growth in the population [7]. While selecting a detrending scheme that maximizes $\bar{r}$ (or EPS) may be appropriate in some circumstances, it is possible that maximising $\bar{r}$ could result in retention of aspects of an ontogenetic trend when many individuals have similar ages and capture years. To help mitigate this risk, spline rigidities that resulted in any non-stationary series, as determined by Kwiatkowski–Phillips–Schmidt–Shin (KPSS) tests ($\alpha = 0.1$) [9,10] were excluded. Following selection of an appropriate detrending scheme, the MIC was recalculated and the cross-dating verification with COFECHA was repeated.

References

1. Holmes, R. L. Computer-assisted quality control in tree-ring dating and measurement. *Tree-Ring Bull.* **43**, 69 - 78 (1983).
2. Matta, M. E., Black, B. A. & Wilderbuer, T. K. Climate-driven synchrony in otolith growth-increment chronologies for three Bering Sea flatfish species. *Mar. Ecol. Prog. Ser.* **413**, 137 - 145 (2010).
3. Black, B. A., Boehlert, G. W. & Yoklavich, M. M. Using tree-ring crossdating techniques to validate annual growth increments in long-lived fishes. *Can. J. Fish. Aquat. Sci.* **62**, 2277 - 2284 (2005).
4. Gillanders, B. M., Black, B. A., Meekan, M. G. & Morrison, M. A. Climatic effects on the growth of a temperate reef fish from the Southern Hemisphere: a biochronological approach. *Mar. Biol.* **159**, 1327 - 1333 (2012).

5. Black, B. A., Boehlert, G. W. & Yoklavich, M. M. Establishing climate–growth relationships for yelloweye rockfish (*Sebastes ruberrimus*) in the northeast Pacific using a dendrochronological approach. *Fish. Oceanogr.* **17**, 368 - 379 (2008).

6. Black, B. A., Allman, R. J., Schroeder, I. D. & Schirripa, M. J. Multidecadal otolith growth histories for red and gray snapper (*Lutjanus spp.*) in the northern Gulf of Mexico, USA. *Fish. Oceanogr.* **20**, 347 - 356 (2011).

7. Wigley, T. M. L., Briffa, K. R. & Jones, P. D. On the average value of correlated time-series, with applications in dendroclimatology and hydrometeorology. *J. Clim. Appl. Meteorol.* **23**, 201 - 213 (1984).

8. von Storch, H. & Navarra, A. (eds) *Analysis of climate variability: applications of statistical techniques*. (Springer, 1999).

9. Kwiatkowski, D., Phillips, P. C. B., Schmidt, P. & Shin, Y. Testing the null hypothesis of stationarity against the alternative of a unit root: how sure are we that economic time series have a unit root? *J. Econometrics* **54**, 159 - 178 (1992).

10. Libanio, G. A. Unit roots in macroeconomic time series: theory, implications, and evidence. *Nova Economia* **15**, 145 - 176 (2005).
